# Drug-Related Disorders and the Criminal and Clinical Background of the Prison Population of São Paulo State, Brazil

**DOI:** 10.1371/journal.pone.0113066

**Published:** 2014-11-19

**Authors:** Maíra Mendes dos Santos, Maria Ines Quintana, Fernanda Gonçalves Moreira, José Geraldo Vernet Taborda, Jair de Jesus Mari, Sérgio Baxter Andreoli

**Affiliations:** 1 Department of Psychiatry, Universidade Federal de São Paulo, São Paulo, São Paulo, Brazil; 2 Program in Public Health, Universidade Catolica de Santos, Santos, São Paulo, Brazil; 3 Department of Clinical Medicine, Universidade Federal do Rio Grande do Sul, Porto Alegre, Rio Grande do Sul, Brazil; Universidade de São Paulo, Brazil

## Abstract

**Objective:**

To analyze the association between drug (DAD) and alcohol (AAD) abuse and dependency and criminal and clinical background by gender of prisoners in São Paulo, Brazil.

**Method:**

Cross-sectional study, random sample stratified by administrative district, from which prisons and prisoners were selected via random, multistage sampling. Psychiatric diagnoses were made with the CIDI 2.1. Lifetime prevalence and 95% CI were calculated and adjusted via analysis of complex samples. Multinomial logistic regression analysis was carried out with four categories of dependent variables: presence AAD; presence DAD; presence of another mental disorder; no mental disorders. For female alcohol and drug abuse and dependency (ADAD) were combined into a single category.

**Results:**

The sample was composed by 1809 interviewed prisoners (1192 men and 617 women). Prevalence of DAD and AAD was 25.2% and 15.6%, respectively, among female prisoners, and 26.5% and 18.5% among males. Male prisoners with DAD were more likely to have a criminal record as an adolescent (OR 2.17), to be a repeat offender (OR 2.85), and to have committed a property crime (OR 2.18). Prisoners with AAD were repeat offenders (OR 2.18). Among female prisoners, ADAD was associated with repeat offenses (OR 3.39), a criminal record as an adolescent (OR 9.24), a clinical or infectious condition (OR 5.09), another health problem (OR 3.04), and violent crime (OR 2.5).

**Conclusion:**

The study confirmed an association between drug-use disorders and the criminal and clinical background in the study population. Prisoners with such disorders were more likely to be repeat offenders and to have a criminal record as adolescents. Among female prisoners disorders were also associated with violent crime and health problems, while among males they were associated with property crime. These patterns in clinical and criminal backgrounds illustrate the need for social rehabilitation programs and specific medical treatment for prison populations.

## Introduction

As a phenomenon that pervades human history, crime has prompted research in a variety of fields, including public health, politics, penal law, and psychiatry. Studies to identify the causes underlying criminal behavior have identified drug-related disorders as a key factor [1]–[5].

Various theoretical models have been put forward to explain the correlation. In general, the relationship may take a direct form (e.g., crimes that involve drugs or crimes committed while under the influence of psychoactive substances) or an indirect form (e.g., because socioeconomic factors put certain groups at high risk for both drug use and crime) [1]; [2]. Although there is so far no comprehensive theoretical model detailing the causal link between these phenomena, studies with prison populations have consistently shown that the relationship is not linear but mediated by several interrelated factors via a complex, multi-causal correlation [3]; [5].

In prison populations worldwide the prevalence of drug-related disorders is very high [6]; [7]. Studies have also shown that prisoners with such disorders tend to have certain criminal and clinical backgrounds. These prisoners face a greater risk of recidivism, behavioral changes while in prison, mental illness [4]; [9] and clinical conditions, such as dermatological, respiratory or infectious and contagious diseases [8].

Understanding the factors involved in the link between these disorders and criminal behavior is important for effective medical and psycho-social interventions, since the lack of adequate intervention in prisons can lead to a vicious cycle of recidivism in drug use, crime, and incarceration [9]; [10]–[13].

Studies of representative samples of the prison population based on standardized psychiatric diagnostic tools remain rare, and this is especially the case for Brazil, which has a large prison population (515000) [14], that is the fourth largest one in the world [15]. The aim of this study was to analyze the relationship between alcohol- and other drug-related disorders and the clinical and criminal background by gender of prisoners in the largest prison population in Brazil, that of São Paulo state. A broader goal was to provide information that could help implement policies to improve public health and reduce criminal recidivism.

## Methods

### Ethics Statement

This study was approved by the ethical committee of the Federal University of Sao Paulo (CEP 1051/05) and the State of Sao Paulo Department of Penitentiary Administration (process n. CS 295/05). All participants signed an informed consent to participate in this study. Individuals that declined to participate were not disadvantaged in any other way by not participating in the study.

### Sample

This was a descriptive, epidemiological study with a cross-sectional design. The sample was representative of the population in the state's 105 prisons (*Unidades Prisonais de regime fechado*): 5 female prisons (PF), 4 female rehabilitation centers (*centros de ressocialização*, CRF), 32 temporary male prisons (*centros de detenção provisória masculinos*, CDP), and 64 male prisons (PM).

Only prisoners housed in prisons were included in the sample, while those housed in maximum-security units or psychiatric hospitals were excluded for reasons of operational difficulty. The study was done between May 2006 and January 2007.

Subjects were selected via random, proportional, multistage sampling. The 5 regional directorates that oversee prisons in the state were treated as strata. For males, 4 prison units were selected at random from each stratum, and prisoners were selected at random from these 20 units (10 PM and 10 CDP). For females, all prisons were included (N = 9) and only prisoners were selected at random (5 PF and 4 CRF). All prisoners were selected at random from a list provided by prison administrators.

The total number of interviews foreseen was 2320, distributed as follows: 1) 690 in PF; 2) 820 in PM; and 3) 810 in CDP. The parameters used for calculation of the sample size are described in Andreoli *et al.* (2014) [16].

The study was approved by the Ethics Committee of the Federal University of São Paulo (Case no. 1051/05) and conducted in accordance with the National Health Council guidelines and standards for the conduct of research involving human subjects (Resolution 196.92). To ensure freedom of consent to all inmates selected for participation in the study, the terms of consent and the resolution 196/92 of the National Health Council were presented to the prison wardens in order to enlighten them that the participation was not mandatory and to ensure that the research did not impose sanctions on those who refused to participate in the study.

### Diagnostic instruments

Data collection was done using the Brazilian version of the Composite International Diagnostic Interview (CIDI), version 2.1 [17]; [18]. The CIDI is a questionnaire that generates psychiatric diagnoses using a computerized algorithm and the criteria of the 10th edition of the *International Classification of Diseases* (ICD-10) and the 4th edition of the American Psychiatric Association's *Diagnostic and Statistical Manual of Mental Orders* (DSM-IV-TR). This study used the ICD-10 criteria.

The following sections of CIDI 2.1 were used in the study: 1) sociodemographic data; 2) anxiety disorders, except for the diagnosis of a specific phobia; 3) depressive disorder and dysthymia; 4) mania; 5) schizophrenia and other psychotic disorders; 6) alcohol-related disorders (alcohol abuse/dependency, AAD); 7) obsessive-compulsive disorder; 8) post-traumatic stress disorder; 9) drug-related disorders (drug abuse/dependency, DAD).

The categories of psychoactive substances included in the study were alcohol, cannabis, stimulants, cocaine, hallucinogens, solvents, sedatives, and opiates.

The questionnaire included questions about criminal background (type of crime, number and length of sentences, criminal record as an adolescent - this variable was scored based on time spent as an adolescent in the rehabilitation system for non-adult offenders, criminal recidivism, etc.), about prison conditions (type of prison; number of prisoners per cell), and about clinical condition, such as dermatological, respiratory and gastric diseases, HIV, STDs, tuberculosis, hepatitis, chronic disease, etc.

The ‘type of crime' variable was scored as one of the following categories: property crimes, drug-related crimes, and violent crimes. The latter category included all crimes that pose a risk to the life of the victim (e.g., armed robbery, rape, homicide, assault, and kidnapping).

### Statistical Analysis

Estimates of the prevalence of AAD and DAD disorders and their respective 95% confidence intervals were calculated and fit to the sampling design using analysis of complex samples [19]. Males and females were analyzed separately due to the different methods of sample selection.

Correlations of the clinical and criminal variables with AAD and DAD were tested using multinomial logistic regression, which is used in cases where the dependent variable is polychotomous [20]. In our study, that variable had four result possibilities: 1) presence of alcohol abuse/dependency (AAD) at some time in life; 2) presence of drug abuse/dependency (DAD) at some time in life; 3) presence of any other mental disorder; 4) no mental disorder at any time. Due to the small size of the female population, the AAD and DAD categories of the dependent variable were combined into a single category, namely ADAD. In our study we analyzed three multinomial logistic regression models for male population and two models for female population, in which we modified the reference category of the dependent variable. For the male population, DAD was compared with ‘no mental disorder’ on the first regression model. On the second model, DAD was compared with ‘other mental disorders’. On the third model, DAD was compared with AAD. For the female population, on the first regression model, ADAD was compared with ‘no mental disorder’. On the second model, ADAD was compared with ‘other mental disorders’.

Regression modeling was done in two steps. In the first step, all independent variables (clinical and criminal factors) correlated with disorders according to a Chi-squared test at a significance level of p<0.10 were included. In the second step, we took into account the Akaike (AIC) and Bayesian information criteria (BIC) generated by the regression analysis [21].

## Results

Interviews were done with 1192 men and 617 women (a 77.9% response rate), which led to sampling loss rates of 26.8% for men and 10.5% for women. The primary reasons for sampling loss were: 1) difficulty accessing selected prisoners; 2) refusals; and 3) transfers between prisons.

### Demographic Data

Most subjects were from São Paulo state, were white, had more than 5 years of schooling, were employed before prison, and had no self-referred health problems. There were differences between genders: most women were single, and they had a larger number of children and poorer-paying jobs before prison than the men. Most individuals attended an educational correctional measure during adolescence due an illicit act, had not been arrested more than once, had been in prison for less than 2 years, were serving 2 criminal sentences. The most common crime category was violent crime, followed by drug-related and property crimes. The prevalence of the crime categories differed between genders. Women committed approximately 4 times more drug-related crimes than men, and men committed more violent crimes. Women were also more likely to be arrested more than once and work more in prison than men (Table 1).

**Table 1:** Social, demographic, clinical, and criminal characteristics of the prison population of São Paulo state, Brazil (N = 1809).

	Feminine Population N (%)	Masculine Population N (%)
**Birthplace**		
São Paulo state	423(68,7)	887(74.4)
Other states	164(26.6)	298(25.0)
Other countries	29(4.7)	7(0.6)
**Race**		
White	338(55.1)	597(51.2)
Black	95(15.5)	222(19.0)
Mixed race	178(29.0)	342(29.3)
Other	2(0.3)	6(0.5)
**Schooling (years)**		
Illiterate	22(3.6)	52(4.4)
0–4	126(20.4)	268(22.5)
5–8	267(43.3)	562(47.3)
More than 8	202(32.7)	307(25.8)
**Worked before going to prison (yes)**	377 (61.1)	932 (78.2)
**Marital status**		
Married	257 (41.9)	704 (59.5)
Not married	356 (58.1)	480 (40.5)
**Number of children**		
None	100 (16.2)	411(34.5)
1–2	274(44.4)	514(43.1)
3–4	174(28.2)	195(16.4)
5 or more	69(11.2)	72(6.0)
**Income**		
No income	252(41.7)	276(23.3)
Up to 1 minimum wage	165(27.3)	231(19.5)
Between 1 and 2 minimum wages	104(17.2)	394(33.3)
More than 2 minimum wages	84(13.9)	282(23.8)
**Works in prison (yes)**	310(50.2)	392(33.0)
**Age (years)**		
18–27	234 (37.9)	581 (48.7)
28–37	222 (36)	381 (32)
38–47	111 (18)	159 (13.3)
48–57	39 (6.3)	56 (4.7)
>57	11 (1.8)	15 (1.3)
**Health problems**		
Clinical infectious illness	142(24.0)	200(18.2)
Other health problems	101(14.5)	134(11.7)
No health problems	340(61.5)	821(70.1)
**Reason for being in prison**		
Drug-related crime	161(26.1)	117(9.8)
Property crime	54(8.8)	84(7.1)
Violent crime	402(65.2)	990(83.1)
**Recidivist** 1	147(24.1)	492(41.6)
	**Feminine Population N (%)**	**Masculine Population N (%)**
**Years of sentence served**		
1 year	121(19.9)	483(41.6)
2 years	181(29.7)	218(18.8)
3 years	132(21.7)	117(10.1)
4 or more years	175(28.7)	343(29.5)
**Criminal record as an adolescent (yes)**	59(9.6)	201(16.9)
**Number of criminal sentences**		
One case	-	-
Two cases	523(84.8)	972(81.8)
Three or more	94(15.2)	216(18.2)

1 Offender arrested more than once

### Prevalence of Alcohol (AAD) and Other Drug (DAD) Abuse/Dependency

Prevalence of DAD and AAD at some time in life (Table 2) were 25.2% and 15.6%, respectively, among women and 26.5% and 18.5% among men. The most common illegal drugs cited for abuse/dependency by both genders were cocaine (women: 17.6%; men: 20.8%), cannabis (women: 8%; men: 12.1%), and sedatives (women: 3.9%; men: 0.6%).

**Table 2:** Lifetime prevalence of mental disorders among the prison population of São Paulo state, Brazil (N = 1809).

	Feminine Population		Masculine Population	
	%	95%IC	%	95%IC
**Alcohol abuse and dependency**	15.6	13.4–18.0	18.5	15.4–22.1
**Drug abuse and dependency**	25.2	19.2–32.2	26.5	22.5–30.8
Opiates	-	-	0.2	0–0.9
Cannabis	8.0	5.2–12.2	12.1	9.0–15.9
Sedatives	3.9	3.5–4.4	0.6	0.2–1.9
Stimulants	1.0	0.6–1.5	0.9	0.4–2.1
Hallucinogens	0.3	0–2.4	0.2	0.1–0.9
Solvents	1.9	1.5–2.4	1.8	1.1–3.1
Other substances	0.2	0–1.6	-	-
**No mental disorder**	20.9	19.6–22.3	38.2	33.5–43.1
**Other mental disorders**	47.5	40.4–54.7	27.2	24.5–30.2

Most prisoners with DAD reported abuse or dependency on just one type of drug (women: 77.1%; IC 67.3–84.6; men: 69.4%; IC 64.4–74.0). By contrast, 83% of women showed comorbidity with other mental disorders (mean of 2 disorders; IC 1.79–2.18), and this was also the case for 64% of men (mean of 1.4 disorders; IC 1.26–1.55). While the rates of comorbidity with abuse and dependency on different types of drugs were similar between genders, comorbidity with other disorders was higher among women.

### Correlations between alcohol (AAD) and other drug (DAD) abuse and dependency and clinical and criminal background

According to the first regression model, men with DAD were more likely than men with no mental disorder to be repeat offenders (OR 2.85), to have a criminal record as an adolescent (OR 2.17), and to have committed a property crime (OR 2.18) (Table 3). Men with DAD were also at greater risk of being repeat offenders than men with other mental disorders (OR 2.08). There were no statistical differences between men with DAD and men with AAD. Men with AAD were more likely to be repeat offenders than men with no mental disorder (OR 2.18) (Table 3) and men with other mental disorders (OR 2.08).

**Table 3:** Associations between abuse and dependency on alcohol and other drugs and clinical and criminal factors among male prisoners compared to prisoners with no mental disorder (N = 1192).

**Drug abuse and dependency**	**P**	**OR**	**95%CI**
Intercept	3.53		
Age	−0.98	0.37	0.17–0.77
Income	−0.25	0.77	0.59–1.02
Clinical/infectious disease^1^	0.35	1.41	0.88–2.26
Other health problems	0.57	1.77	0.97–3.22
Recidivism^2^	1.04	2.85	1.94–4.17
Drug-related crimes	0.52	1.68	0.87–3.22
Property crimes	0.78	2.18	1.11–4.29
Disciplinary problems in prison	0.22	1.25	0.75–2.09
Criminal record as an adolescent	0.77	2.17	1.28–3.68
Married	−0.11	0.89	0.61–1.29
Serving 2 criminal sentences	0.26	1.30	0.80–2.09
**Alcohol abuse and dependency**	**P**	**OR**	**95%CI**
Intercept	−2.17	-	-
Age	0.82	2.27	0.91–5.67
Income	−0.38	0.68	0.47–0.98
Clinical/infectious disease^1^	0.07	1.08	0.54–2.15
Other health problems	0.69	2.01	0.96–4.18
Recidivism^2^	0.78	2.18	1.28–3.71
Drug-related crimes	0.14	1.16	0.44–3.04
Property crimes	−0.12	0.88	0.28–2.76
Disciplinary problems in prison	−0.54	0.58	0.23–1.43
Criminal record as an adolescent	0.35	1.42	0.64–3.15
Married	0.08	1.08	0.64–1.83
Serving 2 criminal sentences	−0.12	0.88	0.47–1.65

^1^ Clinical (e.g., gastric, orthopedic, skin, or respiratory problems, or headaches) and infectious conditions (HIV, tuberculosis, hepatitis, and venereal disease);

2 be arrested more than once. The multivariate logistic regression model included the following categories of dependent variables: ADD, ADA, other mental disorders, and no mental disorder (reference category) Reference categories for independent variables: no health problems; no recidivism; violent crimes; no disciplinary problems; no criminal record as an adolescent; not married; and serving 3 or more sentences (no subject was serving a single sentence).

Women with ADAD were more likely than women with no mental disorder (first model) to be repeat offenders (OR 3.39), have a criminal record as an adolescent (OR 9.24), have a clinical or infectious condition (OR 5.09), have some other health problem (OR 3.04), and have committed a violent crime (OR 2.52) (Table 4).

**Table 4:** Associations between abuse and dependency on alcohol and other drugs and clinical and criminal factors among female prisoners compared to prisoners with no mental disorder (N = 617).

Alcohol and other drug abuse and dependency	P	OR	95%CI
Intercept	0.85		
Age	−0.88	0.41	0.12–1.41
Income	0.28	1.33	0.89–1.99
Clinical/infectious disease^1^	1.62	5.09	2.16–12.01
Other health problems	1.11	3.04	1.29–7.20
Recidivism^2^	1.22	3.39	1.35–8.49
Drug-related crimes	0.62	1.87	0.69–5.06
Violent crimes	0.92	2.52	1.02–6.24
Disciplinary problems in prison	0.11	1.11	0.45–2.77
Criminal record as an adolescent	2.22	9.24	1.11–76.44
Married	−0.04	0.96	0.50–1.82
Serving 2 criminal sentences	−0.06	0.93	0.36–2.42

^1^ Clinical (e.g., gastric, orthopedic, skin, or respiratory problems, or headaches) and infectious conditions (HIV, tuberculosis, hepatitis, and venereal disease); ^2^ be arrested more than once. The multivariate logistic regression model included the following categories of dependent variables: ADD, ADA, other mental disorders, and no mental disorder (reference category) Reference categories for independent variables: no health problems; no recidivism; violent crimes; no disciplinary problems; no criminal record as an adolescent; not married; and serving 3 or more sentences (no subject was serving a single sentence).

Women with ADAD were also more likely than women with other mental disorders (second model) of being repeat offenders (OR 2.19), having a clinical or infectious condition (OR 2.49), and having some other health problem (OR 2.53).

## Discussion

The prison population of São Paulo state shows high prevalence of alcohol- and other drug-related disorders compared to general population [22]. Prisoners with these disorders are more likely to be in prison more than once and to have a criminal record in adolescence. These disorders were also associated among male prisoners with property crimes, and among female prisoners with violent crimes and health problems.

This is the first large-scale epidemiological survey of psychiatric morbidity in the Brazilian prison system to focus on drug-related disorders. It reflects a representative sample of a population that accounts for approximately 40% of all prisoners in Brazil, and is based on standard, proven methods and tools for diagnosing psychiatric conditions. Moreover, the statistical analysis used is an original modeling in this research field and sets our study apart from other studies on this same topic.

Despite its relevance, the study was limited in some respects: 1) prisoner rebellions swept through the prison system a few months before the interviews, which reduced access to some of the selected prisoners; 2) some prisoners may not have provided accurate information regarding drug use because the interviews were carried out inside prisons, where drug possession and use can lead to disciplinary penalties and new criminal charges, and 3) we did not study the social factors that may be important to recidivism, such as family support and structure before and during the sentence, exposure to traumatic events during childhood, and family involvement in crime.

### Prevalence of alcohol (AAD) and other drug (DAD) abuse/dependency

The prevalences of AAD and DAD documented in this study are higher than those in the broader Brazilian population [22], a pattern that has been observed in other countries. While rates of drug-related disorders in prison populations vary between countries, they are generally higher than those we observed [23]–[26]. Explanations for these differences have included the use of different diagnostic tools, cultural differences regarding the criminalization of drug use, and different types of drugs included in the analysis [6].

In our study DAD and AAD rates did not differ between genders. This runs counter to data from the general population, which typically show higher rates among men [22]. High rates of these disorders among female prisoners have also been documented in Australia [27]. Studies have shown that higher rates of DAD among women involved in crime are due to their greater vulnerability to traumatic experiences in life, such as physical, sexual, and domestic abuse and violence [10]; [23]; [26]; [28]; [29]; [30]–[32], as well as family breakdown and the involvement of family members in crime [8]; [33].

Motives for drug consumption tend to differ between genders. For women, drugs may serve as auto-medication to block traumatic feelings and memories and to provide relief from psychological or physical suffering, which is very common to happen among vulnerable population. Men, on the other hand, are motivated to use drugs as a form of curiosity, pleasure-seeking and due to access facilities to drugs [28]; [33].

Both the male and female populations showed the highest lifetime prevalence of abuse/dependence for cannabis, cocaine, and sedatives, in that order. A preference for cannabis and cocaine throughout life has been documented in other studies, both for men [4]; [34] and for women [26]. In a study done in male and female prisons in Portugal, for example, the most commonly used drugs throughout life were cannabis (56%) and cocaine (46%) [2]. In contrast to other studies, opiates were not among the most commonly abused substances in our study [26]; [35]. This may be due to the availability and cost of such drugs in Brazil. Rigorous comparisons with our study are hampered, however, by the fact that other studies only examined drug use and did not diagnose abuse and dependency.

### Factors associated with drug abuse and dependency

Alcohol- and other drug-related disorders were associated with repeat offenses in both the male and female populations. Recidivism rates were significantly higher for these prisoners than for prisoners with no mental disorder and for those with other mental disorders.

A correlation between drug use and recidivism has been reported previously in the literature [3]. A longitudinal study in the United States, for example, found that recidivism rates for prisoners with drug abuse or dependency within one year after release from prison were higher for men (20%) and women (25%) than for prisoners without those disorders (12% M; 9% W) [12].

This recidivism may be explained by a series of factors, including the illegality of drug use, the need to commit thefts in order to purchase drugs, and the social prejudice against drug abuse and dependency. This latter factor makes it harder for released prisoners to rejoin families and the job market, thereby facilitating a return to crime [12].

Our study found that prisoners with DAD and AAD were more likely to have participated in rehabilitation programs related to infractions they committed as adolescents. Entry into crime during adolescence is a well-documented pattern in the literature [4]. The social and psychological stresses common in adolescence (e.g., trouble in school, lack of family support, and personal and social failures) can lead to a vicious cycle of drug use and dependency, which then leads to involvement in crime in order to pay for the dependency [2]; [4]; [36]. In addition, the typical adolescent need to associate and identify with groups may lead a socially vulnerable adolescent who lacks positive role models to join groups involved in drugs and crime [3].

Alcohol- and other drug-related disorders at some point in life showed no association with disciplinary problems in prison. This contrasts with the results of a study of French prisoners, which found that such prisoners were twice as likely to have had disciplinary problems during the previous year [4].

While many characteristics of individuals with DAD and AAD are found in both genders, there are also certain characteristics that are particular to one gender.

### Male population

In the male population we studied, DAD were associated with property crime, as has also been shown by a study in Holland [37]. Dependency on these substances may be directly related to crime because users steal in order to purchase drugs [3], especially for users of cocaine and crack [31], or indirectly related because certain socioeconomic conditions are risk factors for involvement with both drugs and crime [38].

We found no link between violent crime and DAD for men, in contrast to other studies [4]. The same was the case for AAD [39]; [40]. These differing results may be the result of different definitions of violent crime. In our study, armed robbery was included in the violent crime category, while other studies have treated it as property crime. The logic behind our methodological decision was based on the fact that armed robbery in large Brazilian cities has an invariably violent nature [41]. In Brazil, rates of violent crime are very high, reflecting high rates of social inequality [38], powerful drug-trafficking rings, a lack of social capital in poor neighborhoods, and a feeling of impunity among oppressed groups [42]. There are thus many factors apart from drug abuse and dependency that are related to violence in crime, and these may explain the lack of an association between these two factors among Brazilian prisoners.

### Female population

The link between health problems and drug and alcohol-related disorders among female prisoners found in our study has been abundantly documented in the literature [2]; [8]; [11]; [12]; [26]; [29]; [43]. Female prisoners are five times more likely to have health problems than women in the general population [44].

In our study, the strongest association of ADAD was with clinical and contagious conditions. Contagious diseases, such as hepatitis and HIV were also reported as the most common correlates with drug abuse in Spain [26]. The causes underlying this association may be the adverse conditions that these women typically experience, such as prostitution and sexual violence.

The high prevalence of contagious diseases in prisons is especially worrisome because of the potential for transmission to other prisoners, but also because of the potential for transmission to the general population after prisoners are released [2].

ADAD in women was also associated with violent crime. In our study, this correlation may be explained by the high prevalence of comorbidity with other mental disorders. Criminal behavior appears to be associated more closely with mental disorders, which can increase dangerousness level [45].

The negative impact of incarceration on the clinical and social wellbeing of women has been related to their physical and mental vulnerability. Incarcerated women often show auto-destructive behavior such as suicide attempts and a lack of interest in personal health care. Women are also more deeply affected by being far from family, and especially children. For cultural reasons, they are also more susceptible to rejection by society while in prison and after release. All of these factors combine to create and worsen problems with drugs and recidivism [46].

### Issues in the prison system and possibilities for treatment

The large number of prisoners with a history of alcohol- and other drug-related disorders at some point in life and their impact on recidivism have led to debates about the effectiveness of the penal system. Some authors argue that incarcerating these prisoners represents an opportunity for treatment and rehabilitation, given the potential for greater treatment compliance and lower cost. In such programs, treatments typically involve medications and psychotherapy [10]; [47]. Other authors, by contrast, argue that any type of intervention in incarcerated subjects is unsatisfactory, since to be effective a clinical approach should be accompanied by resocialization efforts, such as a reinsertion in the job market and the recovery or rebuilding of social and family ties [11]. Social and psychological support increases subjects' motivation for facing dependency and encourages them to respect societal requirements and thereby maintain pro-social, non-deviant behavior [1]. This type of approach is typically expressed via alternative sentencing.

Judicial options for alternative sentencing that do not require incarceration, depending on the severity of the disorder and the crime committed, have been established in some countries and have an increasingly firm foundation in law. Interest in alternative sentencing is on the rise because it recognizes the specific needs that prisoners with alcohol- and other drug-related disorders have for medical and psychosocial interventions [3]. Brazilian law, for example, allows sentences in certain cases based on stays in therapeutic facilities (*comunidades terapêuticas*) or community service. This legislation is not entirely effective, however [48], mostly due to a lack of criminal investigators needed to carry out clinical, psychiatric, and psychological evaluations of offenders.

This shortcoming means that many individuals with alcohol- and other drug-related disorders end up in regular prisons, where specialized support for the treatment they need is absent or poor [49]. While Brazil's National Health Plan for the Prison System includes actions to promote medical and psycho-social well-being via treatment and prevention in the prison and public health systems, there are no programs focused on the prevention and treatment of alcohol- and other drug-related disorders [50].

There is also a shortage of clinics for medical care, as well as general overcrowding and unhealthy conditions in prison facilities [51]. Shortages of health care professionals who can provide and track medical and psychological treatment in the prison system are a common problem in Brazil. In the case of São Paulo, Brazil's National Department of Prisons [14] indicates that there are 35 psychiatrists, 56 health clinics, and 183 psychologists for 100,000 prisoners. Given the high prevalence of mental disorders in prisons [52] and the added difficulty of treating patients in a prison context, this number of health professionals is far from sufficient. Given the urgent and specific needs of prisoners with drug-related disorders, the current situation remains discouraging.

## Conclusion

This study has shown that alcohol- and other drug-related disorders are prevalent and related to educational correctional measure during adolescence and recidivism during adulthood for both male and female prisoners. The type of crimes committed varied with gender; men with drug abuse and dependency committed more property crimes, while women with alcohol- and other drug-related disorders committed more violent crimes. Women with these disorders were also prone to health problems.

Our findings suggest that prisoners with alcohol- and other drug-related disorders have specific clinical needs and criminal background, so it should be considered during the execution of the sentence. Therefore, understanding patterns in the clinical and criminal background of this subpopulation is important because it can contribute to the effective implementation of prison policies regarding the prevention and treatment of drug use. In turn, this will help fulfill the primary mission of the prison system, which is social rehabilitation and the reduction of recidivism.
